# Which machine learning algorithms are best at predicting eating disorder tendencies among nursing students in China?

**DOI:** 10.1186/s12912-026-04951-y

**Published:** 2026-06-29

**Authors:** Zenghui Wang, Chen Chen, Xiangjie Sun, Tiantian Deng, Deyan Yang, Danjun Feng

**Affiliations:** 1 School of Nursing and Rehabilitation, Cheeloo College of Medicine, Shandong University, Wenhuaxi Road 44, Jinan, Shandong 250012 China; 2 Department of Orthopedic Surgery, Qilu Hospital of Shandong University, Wenhuaxi Road 107, Jinan, Shandong 250012 China; 3 Department of Minimally Invasive Thoracic Surgery and Lung Transplantation, Shandong Provincial Hospital Affiliated to Shandong First Medical University, Jinan, Shandong 250021 China; 4 Department of Interventional Division, Shandong Cancer Hospital and Institute, Shandong First Medical University and Shandong Academy of Medical Sciences, Jinan, Shandong 250117 China

**Keywords:** Eating disorder, Machine learning, Nursing students, Random forest, Nomogram

## Abstract

**Background:**

Nursing students are at high risk for developing eating disorders (ED). However, methods for identifying ED tendencies within this group remain underexplored.

**Purpose:**

This study aims to develop and validate machine learning-based models for predicting ED tendencies among nursing students.

**Methods:**

A cross-sectional study was conducted with a sample of nursing students in mainland China (*N* = 1709). Machine learning models were developed using a training set (*N* = 1196), and internally validated using a test set (*N* = 513). Model performance was evaluated based on widely used metrics, including the area under the receiver operating characteristic curve (AUC).

**Results:**

The random forest performed best in test set, achieving a Bootstrapped_AUC of 0.804 (95% CI, 0.787, 0.818), a Bootstrapped_AP of 0.711 (95% CI, 0.682, 0.739), and a brier score of 0.170. The non-black box models showed that rumination related to ED, low body satisfaction, and depression were significant risk factors of eating disorders tendencies among nursing students.

**Conclusion:**

Machine learning-based models can help nursing educators to identify nursing students at risk of ED. Additionally, addressing cognitive biases regarding body shape and weight, as well as alleviating depressive symptoms, may help reduce ED risk among nursing students.

**Clinical trial number:**

Not applicable.

**Supplementary Information:**

The online version contains supplementary material available at https://doi.org/10.1186/s12912-026-04951-y.

## Introduction

Eating disorders (ED) is defined as a group of psychiatric disorders (e.g., anorexia nervosa, bulimic nervosa, and binge eating disorder) characterized by abnormal eating behaviors, and excessive concerns about food, weight, and body shape, according to the *DSM-5* [1]. ED currently accounted for a fifth of psychiatric diseases in China [2], and mainly occurred in the youth, including college students, with a lifetime prevalence of 35.0% [3, 4].

Epidemiological surveys on ED have shown that being female, having low body satisfaction, poor mental health, academic stress, and unhealthy eating behaviors are primary risk factors for ED among college students. Previous studies have found that these risk factors are particularly prevalent in nursing students. First, in Southeast Asian countries, women comprise approximately 89.0% of the nursing workforce [5]. Second, Blake [6] founds that 78.0% of nursing students in South Korea had low body satisfaction. Third, the meta-analyses showed that depression disorder had higher prevalence in Aisa nursing students (43.0%) than general college students (33.6%) [7, 8]. Fourth, a meta-analysis by Zheng [9] founds that 65.2% of nursing college students had moderate-to-high perceived stress levels. Lastly, 18.0% of Chinese nursing students resort to unhealthy weight-loss methods, such as dieting and laxative use [10]. These factors suggest a potentially higher ED risk among nursing students. However, research on ED prevalence within this population remains limited. Only two studies investigated the ED status in nursing students and reported that 28.4% of India nursing students had higher ED risk [11], and 73.5% of Turkey nursing students was in the risk of orthorexia nervosa (i.e., one subtype of ED) [12].

Previous studies have highlighted that ED exert numerous negative effects on the physical, mental, and social well-being of nursing students. Enormous studies indicated that college students with ED often suffered from cardiovascular, digestive, and endocrine diseases, etc [13]. ED also increase their risk for other psychiatric disorders, such as depression and obsessive-compulsive disorder, and are associated with self-harm and suicidal behaviors [14]. Furthermore, ED impair their academic performance and hinder social interaction skills [14–16]. Moreover, some studies suggest that these ED-related health issues, including physical ailments and depression, may lead to increased turnover among newly graduated nurses, exacerbating the shortage of nursing professionals [17]. Therefore, early detection of ED tendencies among nursing students is essential.

Most studies have used logistic regression to develop early warning models for ED. For instance, Li identified anxiety, emotional and physical neglect, and being overweight or obese as primary risk factors for ED among junior school students [18]. Similarly, Aparicio-Martinez et al. [19] found that body dissatisfaction, ideal body shape, low self-esteem, and excessive media use were significant risk factors for ED in female undergraduate students, with the logistic regression model achieving an AUC of 0.94 in the original sample. However, these studies often construct ED models from a specific research perspective, which may limit their predictive accuracy on external samples. McCabe et al. [20] proposed the theoretical model of ED risk and protective factors based on comprehensive literatures review, categorizing influencing factors into five domains: demographic (e.g., gender, age, and educational level), physical (e.g., BMI, and medical condition), psychological (e.g., perfectionism, impulsivity, and body dissatisfaction), behavioral (e.g., physical exercise, and emotional eating), and social factors (e.g., experiences of trauma or stress). This model thoroughly describes the mechanisms linking ED to each type of influencing factor, suggesting that ED arise from a complex interplay of multiple factors. Although this model covered most of factors of ED from multiple perspectives, it still ignored the factors from family perspective led to ED. Spanou et al. [21] summarized family factors affecting ED and divided these factors into two aspects including general family factors and ED specified family factors. The general family factors included family structure, history of childhood maltreatment, and family relationships. The ED-specified family factors contained family eating environment and family members attitude towards eating, body shape, and weight. Some studies indicated that parents’ BMI could reflect their family eating environment and attitude to eating and body shape. Parents with obesity or overweight could predict their children’s poor eating behaviors [22].

With the rise of artificial intelligence, ML studies on ED have increased significantly, reaching peak of more than 20 publications in 2023 [23]. Current ML research on ED remains predominantly centered on diagnosis and prognosis prediction of the disorder. Clinical studies have largely used structural MRI, functional MRI for ED diagnosis [24]. Several prognostic studies have compared traditional models and ML models, demonstrating that ML approaches have advantages in prediction tasks. For example, Haynos et al. [25] found that in the task of predicting ED patients’ disease progression, such as binge eating and compensatory behaviors at the follow-up 1 and 2 years, ML outperformed traditional logistic regression, achieving an AUC of 0.78 compared to 0.67. Similarly, a randomized controlled trial on ED patients indicated that ML yielded better predictive accuracy than logistic regression for treatment outcomes, including binge eating abstinence (AUC = 0.51 vs. 0.50) and weight loss (AUC = 0.68 vs. 0.66) [26].

Compared to the extensive research on ED diagnosis and prognosis, screening studies on ML remain scarce. Existing screening research has primarily focused on using eating-related psychological and behavioral indicators to identify individuals at high risk for ED or for their initial onset. Ren et al. [27] incorporated BMI, ED-related psychological (e.g., eating inflexibility, body image inflexibility, body dissatisfaction) and behavioral measures (e.g., emotional overeating, loss of control over eating) into a decision tree model to screen for high-risk ED in students female, achieving favorable predictive performance with a sensitivity of 0.88 and specificity of 0.85. Stice and Desjardins [28] applied a decision tree model to identify ED onset in high-risk females. Their study further expanded the range of predictors beyond BMI, eating-related psychological (e.g., thin-ideal internalization, thinness expectancy, body dissatisfaction) and behavioral factors (e.g., weight control behaviors, dieting) to include impaired social function and mental health utilization. Notably, these studies relied on a single model without exploring optimal model selection.

ML encompasses various algorithms, including Lasso regression, Ridge regression, support vector machines (SVM), random forest (RF), and artificial neural networks (ANN), each with distinct advantages. Lasso and Ridge regression are well-suited for linear problems and produce interpretable results, whereas ANN is better for handling complex nonlinear problems but operates as a “black box” [29]. According to the “No Free Lunch” theorem, no single model can be deemed universally optimal for predictive tasks, such as disease screening [30]. Thus, comparing the predictive performance of these algorithms is essential to determine the most suitable model for identifying ED tendencies among nursing students. Accordingly, the present study was designed with the following objectives:

1. Targeting nursing students, a population at potentially high risk for ED;
2. Incorporating more comprehensive predictors, including physiological, psychological, and social factors, to improve predictive performance;
3. Conducting multiple ML models comparisons to identify the optimal model;
4. Using questionnaire-based assessments, making the resulting model more convenient, cost-effective, and practical for real-world use.

## Methods

### Participants

The cross-sectional study was conducted between March and April 2022 in mainland China. The inclusion criteria were as follows: (1). participants were 16 years or above; (2). participants were full-time nursing college students; (3). their participation was voluntary. The exclusion criteria were as follows: (1). participants were suspended from school for three months or more; (2). participants were diagnosed with serious physical or psychiatric disorders. The nursing students were surveyed using “Wenjuanxing”, an online data collecting tool, through their online class group in Wechat or QQ software and given gifts as compensation. This study used a convenience sampling method to collect 1,856 respondents’ data and finally included 1,709 respondents’ questionnaires, representing a valid 92.08% response rate. The sample contained 1,243 females (72.7%) and 466 males (27.3%) aged between 16 and 30 years (M = 21.13, SD = 1.80). The detailed sample characteristics were presented in Table 1.

**Table 1:** Univariate analysis of eating disorder tendencies among nursing students in China (*N* = 1709)

Variables	Total	None Eating Disorders	Eating Disorders	Statistical Value	*P*-value
	*N* (%) / M ± SD / MD (IQR)	*N* (%) / M ± SD / MD (IQR)	*N* (%) / M ± SD / MD (IQR)	χ^2^/T/F/Z	
***Total***	1709 (100)	1035 (60.6)	674 (39.4)		
***Demographics***					
Age (Years)				3.317	0.069
≤ 21	1037 (60.7)	646 (62.3)	391 (37.7)		
> 21	672 (39.3)	389 (57.9)	283 (42.1)		
Gender				4.100	0.043
Male	466 (27.3)	264 (56.7)	202 (43.3)		
Female	1243 (72.7)	771 (62.0)	472 (38.0)		
Ethnicity				1.486	0.223
Han	1661 (97.2)	1010 (60.8)	651 (39.2)		
Minority	48 (2.8)	25 (52.1)	23 (47.9)		
Religious Beliefs				15.384	**< 0.001**
Yes	102 (6.0)	43 (42.2)	59 (57.8)		
No	1607 (94.0)	992 (61.7)	615 (38.3)		
Residence				9.283	0.002
Rural	615 (36.0)	402 (65.4)	213 (34.6)		
Urban	1094 (64.0)	633 (57.9)	461 (42.1)		
CSUL				35.490	**< 0.001**
Megacity	364 (21.3)	182 (50.0)	182 (50.0)		
Super city	937 (54.8)	624 (66.6)	313 (33.4)		
Type I large city	199 (11.6)	115 (57.8)	84 (42.2)		
Type II large city	141 (8.3)	79 (56.0)	62 (44.0)		
Medium and small city	68 (4.0)	35 (51.5)	33 (48.5)		
Education Level				14.932	**0.001**
Junior college	237 (13.9)	125 (52.7)	112 (47.3)		
Undergraduate	1329 (77.8)	806 (60.6)	523 (39.4)		
Postgraduate	143 (8.4)	104 (72.7)	39 (27.3)		
Grade				6.977	0.073
Freshman	386 (22.6)	253 (65.5)	133 (34.5)		
Sophomore	601 (35.2)	358 (59.6)	243 (40.4)		
Junior	567 (33.2)	340 (60.0)	227 (40.0)		
Senior	155 (9.1)	84 (54.2)	71 (45.8)		
Single Status				3.706	0.054
Yes	1226 (71.7)	760 (62.0)	466 (38.0)		
No	483 (28.3)	275 (56.9)	208 (43.1)		
***Biological Factors***					
HPI				1.041	0.308
Yes	1687 (98.7)	1024 (60.7)	663 (39.3)		
No	22 (1.3)	11 (50.0)	11 (50.0)		
BMI				14.163	**0.001**
Thin	388 (22.7)	234 (60.3)	154 (39.7)		
Norm	1093 (64.0)	688 (62.9)	405 (37.1)		
Overweight or obesity	228 (13.3)	113 (49.6)	115 (50.4)		
Insomnia		3.00 (2.00, 6.00)	5.00 (3.00, 9.00)	11.719	**< 0.001**
***Psychological Factors***					
Introversion	5.30 ± 1.701	5.21 ± 1.745	5.44 ± 1.622	-2.738	0.006
Neuroticism	4.84 ± 1.618	4.60 ± 1.537	5.20 ± 1.674	-7.550	**< 0.001**
Conscientiousness	6.94 ± 1.641	7.13 ± 1.601	6.64 ± 1.660	6.070	**< 0.001**
Perfectionism	23.65 ± 5.697	22.31 ± 5.597	25.71 ± 5.218	-12.592	**< 0.001**
Impulsivity	16.18 ± 4.546	15.39 ± 4.459	17.40 ± 4.410	-9.141	**< 0.001**
Anxiety	5.00 (1.00, 8.00)	3.00 (0.00, 7.00)	7.00 (4.00, 11.0)	15.489	**< 0.001**
Depression	5.00 (2.00, 10.0)	4.00 (1.00, 7.00)	9.00 (4.00, 14.00)	16.088	**< 0.001**
Loneliness	4.00 (3.00, 6.00)	4.00 (3.00, 5.00)	5.00 (4.00, 6.00)	11.098	**< 0.001**
Body Satisfaction	34.60 ± 7.286	35.25 ± 7.222	33.59 ± 7.275	4.616	**< 0.001**
RED	20.19 ± 6.773	17.86 ± 6.528	23.76 ± 5.464	-19.442	**< 0.001**
***Behavioral Factors***					
Exercise				0.351	0.839
Little	1193 (69.8)	724 (60.7)	469 (39.3)		
Moderate	396 (23.2)	236 (59.6)	160 (40.4)		
Vigorous	120 (7.0)	75 (62.5)	45 (37.5)		
BCAB	12.00 (9.00, 15.00)	11.0 (9.00, 14.00)	14.00 (11.00, 17.00)	12.174	**< 0.001**
Emotional Eating	9.19 ± 3.017	8.54 ± 3.062	10.17 ± 2.663	-11.299	**< 0.001**
TSMM				12.143	0.002
<15 min	943 (55.2)	543 (57.6)	400 (42.4)		
15–30 min	689 (40.3)	451 (65.5)	238 (34.5)		
>30 min	77 (4.5)	41 (53.2)	36 (46.8)		
FHCC	2.85 ± 0.978	2.70 ± 0.943	3.08 ± 0.986	-8.185	**< 0.001**
FVV	3.89 ± 0.850	4.01 ± 0.817	3.72 ± 0.869	6.958	**< 0.001**
FRM	3.98 ± 0.896	4.09 ± 0.869	3.82 ± 0.914	6.106	**< 0.001**
CFBM	3.04 ± 0.949	2.92 ± 0.946	3.22 ± 0.924	-6.577	**< 0.001**
***Family Factors***					
Two-parent Upbringing				4.760	0.029
Yes	1572 (92.0)	964 (61.3)	608 (38.7)		
No	137 (8.0)	71 (51.8)	66 (48.2)		
Only Child				4.580	0.032
Yes	851 (49.8)	537 (63.1)	314 (36.9)		
No	858 (50.2)	498 (58.0)	360 (42.0)		
MCE				5.881	0.053
<1000 CNY	368 (21.5)	243 (23.4)	125 (18.5)		
1000–2000 CNY	966 (56.5)	571 (55.1)	395 (58.6)		
>2000 CNY	375 (21.9)	221 (21.3)	154 (22.8)		
Father’s Education Level				4.551	0.208
Primary school or below	144 (8.4)	87 (60.4)	57 (39.6)		
Junior high school	532 (31.1)	341 (64.1)	191 (35.9)		
Senior high school	690 (40.4)	401 (58.1)	289 (41.9)		
College or above	343 (20.1)	206 (60.1)	137 (39.9)		
Father’s BMI				7.071	0.029
Thin	200 (11.7)	108 (54.0)	92 (46.0)		
Norm	1263 (73.9)	788 (62.4)	475 (37.6)		
Overweight or obesity	246 (14.4)	139 (56.5)	107 (43.5)		
Father’s HPI				1.283	0.257
Yes	134 (7.8)	75 (56.0)	59 (44.0)		
No	1575 (92.2)	960 (61.0)	615 (39.0)		
Mother’s Education Level				7.922	0.048
Primary school or below	270 (15.8)	174 (64.4)	96 (35.6)		
Junior high school	552 (32.3)	348 (63.0)	204 (37.0)		
Senior high school	608 (35.6)	342 (56.3)	266 (43.8)		
College or above	279 (16.3)	171 (61.3)	108 (38.7)		
Mother’s BMI				4.738	0.094
Thin	202 (11.8)	112 (55.4)	90 (44.6)		
Norm	1295 (75.8)	803 (62.0)	492 (38.0)		
Overweight or obesity	212 (12.4)	120 (56.6)	92 (43.4)		
Mother’s HPI				0.480	0.488
Yes	108 (6.3)	62 (57.4)	46 (42.6)		
No	1601 (93.7)	973 (60.8)	628 (39.2)		
Childhood Adversity				106.321	**< 0.001**
Yes	443 (25.9)	177 (40.0)	266 (60.0)		
No	1266 (74.1)	858 (67.8)	408 (32.2)		
***Social Factors***					
SSES	5.64 ± 1.839	5.57 ± 1.745	5.74 ± 1.971	-1.872	0.061
Social Support	10.0 (9.00, 12.00)	11.0 (9.0, 12.0)	10.00 (9.00, 11.0)	-6.639	**< 0.001**
Perceived Stress	4.00 (3.00, 6.00)	4.00 (2.00, 5.00)	5.50 (4.00, 7.00)	13.094	**< 0.001**
Traumatic Events				36.504	**< 0.001**
Yes	177 (10.4)	70 (39.5)	107 (60.5)		
No	1532 (89.6)	965 (63.0)	567 (37.0)		

NOTES: N: Frequency; M: Mean; SD: Standard Deviation; MD: Median; IQR: Interquartile Range; CSUL: City Size of University’s Location; HPI: History of Present Illnesses; BMI: Body Mass Index; RED: Rumination related to Eating Disorders; BCAB indicates Body Checking and Avoiding Behavior; TSMM: Time Spent on Meals; FHCC: Frequency of High Caloric Food Consumption; FVC: Frequency of Vegetables Consumption; FRM: Frequency of Regular Meals; CFBM: Consumption of Food Between Meals; MCE: Monthly Consumption Expenditure; CNY: Chinese Yuan; SSES: Subjective Social and Economic Status; Categorical variables use column percentages in the total sample and row percentages in the subsample (None-eating disorders or eating disorders)

### Ethical considerations

This study adhered to the principle of the Declaration of Helsinki. Ethical approval for this study was obtained from the Ethics Committee of the School of Nursing and Rehabilitation, Shandong University (Approval No. 2017-R-102). Informed consent was collected from all participants. Additionally, for students under the age of 18, informed consent was also obtained from their guardians.

### Measurements

The Sick, Control, One Stone, Fat, Food (SCOFF) questionnaire [31] was used for outcome measurements. This scale comprises five items of core symptoms of anorexia nervosa and bulimic nervosa, using the scoring method of “0 = no” and “1 = yes”. A total score of 2 points or above was identified as a positive sample, while others were identified as negative.

The predictor measurements included demographic, biological, psychological, behavioral, family, and sociological data. The demographic questionnaire included age, gender, nationality, religious belief, only child status, household, partner situation, education level, university city, and grade (see details in Table 1). The biological questionnaire included variables of history of present illness, insomnia, and BMI. The psychological questionnaire included variables of introversion, neuroticism, conscientiousness, impulsiveness, perfectionism, anxiety, depression, and loneliness, rumination, and body satisfaction. The behavioral questionnaire included variables of eating habits, emotional eating, body checking and avoidance behavior, and physical exercise. The family questionnaire included variables of family pattern, childhood adversity, parent’s BMI, and parent’s educational level. The sociological questionnaire included variables of monthly consumption expenditure, socioeconomic status, social support, traumatic events, stress, and subjective academic performance. Specifically, some variables used one item to reflect, while others used standard scale to measure. The scales’ reliability, validity, and references were presented in Supplementary Table 1. Cronbach’s α ≥ 0.70 is adequately acceptable, 0.60–0.70 marginally acceptable but requires cautious interpretation, < 0.60 unacceptable [32]. Most scales met the minimum acceptable requirement (Cronbach’s α = 0.700–0.940), except the SCOFF (0.536), the Activity Rating Scale (0.626), and two-item personality questionnaires (0.281–0.344). The SCOFF, a widely used ED screening tool, typically showed lower Cronbach’s α due to low inter-item correlations among its five core items, and was generally reported between 0.40 and 0.60 in previous studies [33, 34]. The two-item personality questionnaire’s low Cronbach’s α was due to its limited items and reverse scoring [35, 36]. Finally, we retained the Activity Rating Scale given its theoretical relevance to eating disorders [37]; accordingly, we caution against over-interpretation of these results.

### Statistical analysis

All statistical analysis was performed in R (4.0.5) and Python (3.7.9).

Feature variable (predictor) selection was performed prior to ML model construction. Some variables with minimal or no statistical impact on the outcome variable may introduce noise during model training, potentially compromising model fit. To construct a concise dataset with minimal noise, univariate analyses (e.g., t-test, Mann-Whitney U test, and Chi-square test) and two-way stepwise logistic regression (LR) were conducted using the *glm* package in R 4.0.5. Bonferroni correction was applied to control the risk of type I error inflation due to multiple hypothesis testing. When 7 or more independent hypotheses are tested simultaneously at the same dataset, the *P* value of each hypothesis should be the one out of n (i.e., inverse number of hypothesis) of the *P* value of one independent hypothesis [38]. Given that this study tested 44 independent variables, the corrected *P* value threshold was set at 0.001. Additionally, the multicollinearity diagnosis (VIF < 5) was performed to ensure the validity of two-way stepwise LR. Due to different scales of selected independent variables from univariate analysis (*P* < 0.001), this study normalized the independent variables to better observe the impact of them on ED tendencies.

The original set (*N* = 1709) was randomly divided into a training set (*N* = 1196), and a test set (*N* = 513) in a 7:3 ratio. The training set was used for hyperparameter optimization and models construction, and the test set for model fit testing and models comparison. The ML models training was performed by *Scikit-Learn* package 1.0.2. in Python 3.7.9. The algorithms used in this study included Lasso, Ridge, SVM, RF, and ANN. The 10-fold validation and grid search were used to find the optimal hyperparameters.

The ML models were evaluated by using the AUC, which ranged from 0.50 (no discrimination) to 1.00 (perfect discrimination). The guidelines of AUC scored poor (< 0.70), fair (0.70–0.79), good (0.80–0.89) and perfect (≥ 0.90). To exactly assess predictive accuracy, the average AUC and its 95% confidence interval (CI) in the test set were calculated using 1,000 bootstrapped resamples. In addition to AUC, this study also reports average precision (AP), accuracy (ACC), sensitivity, specificity, precision, negative predictive value (NPV), F1 score, Brier score, and the Youden index as supplementary metrics. Although a comprehensive explanation of these ML models is beyond this paper’s scope, readers are referred to Kuhn and Johnson [39] for an in-depth introduction to these models and general ML concepts.

As artificial neural networks (ANN) typically require larger sample sizes than other algorithms, we calculated the minimum sample size necessary for ANN in this study. We employed the direct empirical method, setting the minimum sample size at ten times the total number of network weights (ω). The ANN structure used here consisted of an input layer (N), one hidden layer (H), and an output layer (M). Following Hinton et al. [40], one of the recommended numbers of hidden neurons is 2/3 of the input neurons, plus the size of the output neurons. This study presets input layer neurons as 10, resulting in an ANN with 10 input neurons, 2 output neurons, and 9 hidden neurons. The total network wight was calculated as 108 (ω = M×H + N×H), setting the minimum sample size at 1080. Considering the 10.0% invalid questionnaires, the final sample sizes 1200. This study thus meets the simple size requirements.

## Results

### Univariate analysis

According to Table 1, the dependent variable of ED tendencies (0 = “non-eating disorder” and 1 = “eating disorder”) and forty-four independent variables were included in univariate analysis. The results showed that twenty-four independent variables (*P* < 0.001) were totally included for the following multivariate logistic regression, containing religious beliefs, CSUL, educational level, BMI, insomnia, neuroticism, conscientiousness, perfectionism, impulsivity, anxiety, depression, loneliness, body Satisfaction, RED, BCAB, emotional eating, FHCC, FVC, FRM, CFBM, childhood adversity, social support, perceived stress, and traumatic events.

### Stepwise multivariate logistic regression

Table 2 showed the two-way stepwise multivariate LR model with adjusted OR and 95% CI of the ten independent variables, which were screened as the ML’s feature dataset. According to the impact of influencing factors on ED tendencies, the strongest predictor was RED (OR = 32.680, *P* < 0.001), followed by body satisfaction (OR = 0.306, *P* = 0.001), depression (OR = 2.139, *P* = 0.033), perceived stress (OR = 2.106, *P* = 0.010), FVC (OR = 0.477, *P* = 0.010), childhood adversity (OR = 1.678, *P* < 0.001), religious beliefs (OR = 1.673, *P* = 0.041), and overweight or obesity of BMI (OR = 1.630, *P* = 0.015).

Although insomnia (OR = 1.959, *P* = 0.086) and emotional eating (OR = 1.595, *P* = 0.080) did not reach statistical significance at the conventional level of *P* < 0.05, they were retained in the final model based on a predefined selection criterion of *P* < 0.10, as well as their clinical predictive purposes and potential role in confounding adjustment.

**Table 2:** Multivariate logistic regression of eating disorder tendencies among nursing students in China (*N* = 1709)

Variables	B	S.E.	Wald	OR	95% C.I.	*P* Value
Constant	-1.840	0.354	26.953	0.159		< 0.001
Religious Beliefs	0.515	0.252	4.185	1.673	1.022–2.739	0.041
BMI			8.373			0.015
Norm	0.002	0.144	0.000	1.002	0.756–1.327	0.992
Overweight or obesity	0.489	0.200	5.948	1.630	1.101–2.414	0.015
Insomnia	0.673	0.392	2.948	1.959	0.909–4.221	0.086
Depression	0.760	0.356	4.562	2.139	1.065–4.299	0.033
Body Satisfaction	-1.184	0.356	11.036	0.306	0.152–0.615	0.001
RED	3.487	0.298	136.932	32.68	18.225–58.603	< 0.001
Emotional Eating	0.467	0.267	3.068	1.595	0.946–2.689	0.080
FVC	-0.740	0.288	6.583	0.477	0.271–0.840	0.010
Childhood Adversity	0.518	0.145	12.802	1.678	1.264–2.228	< 0.001
Perceived Stress	0.745	0.289	6.646	2.106	1.196–3.711	0.010
Nagelkerke R^2^	0.347					

NOTES: No of Religious Beliefs, Thin of BMI, No of Childhood Adversity are control group; BMI: Body Mass Index, RED: Rumination related to Eating Disorders, FVC: Frequency of Vegetables Consumption; All independent variables were normalized to range [0, 1]

### ML model performance

Supplementary Table 2 presents the optimal hyperparameters and AUC values for each ML model. No overfitting was observed across the models, as evidenced by the similar AUC scores in both the training and test sets. For instance, the AUC for the RF model was 0.808 in the training set and 0.818 in the test set. The closeness of these AUC scores indicates that the RF model did not experience overfitting or underfitting issues.

Table 3 summarized the predictive performance metrics for each ML models in training set and testing set. This study computed some indicators through confusion matrix, including accuracy, specificity, sensitivity, precision, NPV and F_1_ score. These metrics evaluate the difference between the true values and the predicted values. The predicted values were classified as positive (≥ 0.5) or negative (< 0.5) using a default threshold of 0.5. It is important to note that these metrics are sensitive to threshold changes; adjusting the threshold would alter the prediction values. These metrics are considered reference points rather than robust measures of model performance. This study used the default threshold (0.5) instead of the optimal threshold (Youden index), as the latter varied across the five models and did not provide a consistent standard.

**Table 3:** Model performance in predicting eating disorders tendencies in the training (*N* = 1196) and Test Set (*N* = 513)

	AUC	Bootstrapped_AUC (95% CI)	AP	Bootstrapped_AP (95% CI)	ACC	Sensitivity	Specificity	Precision	NPV	F1	Brier	Yorden
Training set												
Lasso	0.798	0.801 (0.797, 0.804)	0.706	0.703 (0.693, 0.710)	0.745	0.616	0.830	0.704	0.767	0.657	0.175	0.446
Ridge	0.799	0.802 (0.798, 0.804)	0.705	0.702 (0.694, 0.708)	0.738	0.612	0.821	0.692	0.763	0.649	0.176	0.433
RF	0.808	0.917 (0.905, 0.927)	0.936	0.882 (0.859, 0.900)	0.877	0.806	0.924	0.874	0.879	0.839	0.105	0.730
SVM	0.798	0.801 (0.794, 0.804)	0.700	0.697 (0.687, 0.704)	0.742	0.584	0.846	0.714	0.756	0.643	0.176	0.431
ANN	0.799	0.802 (0.798, 0.804)	0.702	0.699 (0.689, 0.707)	0.744	0.612	0.831	0.704	0.765	0.655	0.175	0.443
Test set												
Lasso	0.793	0.792 (0.782, 0.800)	0.719	0.715 (0.700, 0.725)	0.715	0.565	0.812	0.657	0.745	0.608	0.177	0.377
Ridge	0.798	0.794 (0.784, 0.801)	0.717	0.714 (0.702, 0.723)	0.717	0.580	0.805	0.655	0.750	0.615	0.177	0.385
RF	0.818	0.804 (0.787, 0.818)	0.731	0.711 (0.682, 0.739)	0.749	0.630	0.824	0.696	0.777	0.661	0.170	0.454
SVM	0.795	0.792 (0.776, 0.801)	0.716	0.710 (0.694, 0.721)	0.727	0.550	0.840	0.688	0.745	0.611	0.178	0.390
ANN	0.795	0.793 (0.783, 0.801)	0.717	0.713 (0.700, 0.723)	0.727	0.580	0.821	0.674	0.754	0.624	0.178	0.401

NOTES: RF: Random Forest; SVM: Support Vector Machine; ANN: Artificial Neural Network; AUC: Area Under Curve of ROC; AP: Average Precision; ACC: Accuracy; NPV: Negative Predictive Value. AUC and AP are derived from the raw data set outputs, while Bootstrapped_AUC and Bootstrapped_AP are estimated using the bootstrap method in which the data set has been resampled 1000 times

**Fig. 1:**
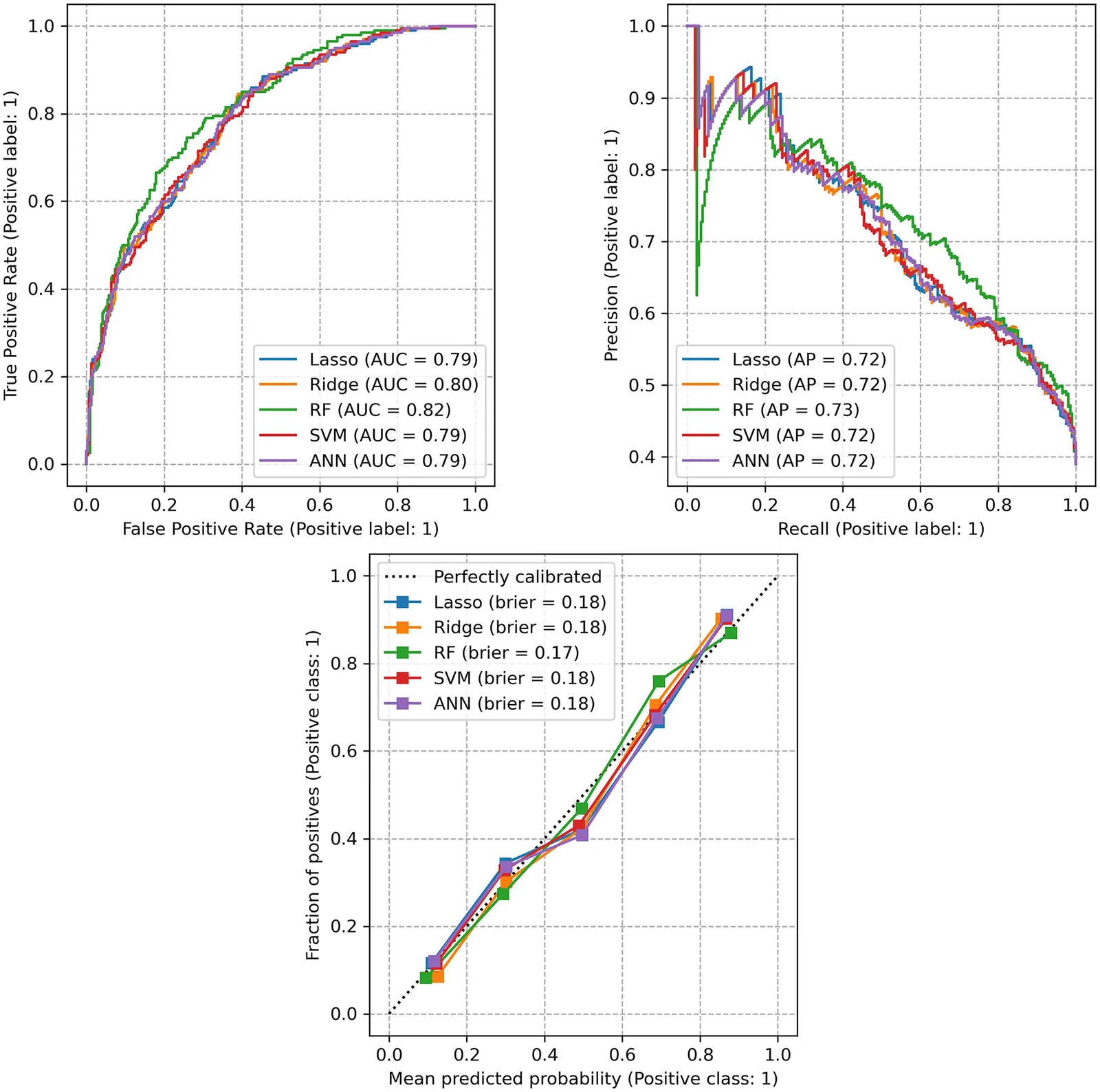
ROC curve, PR curve, and calibration plot of different ML models in the test set. (A represents the ROC curve. B represents the PR curve. C represents the calibration plot. RF: random forest; SVM: support vector machine; ANN: artificial neural network). The value of AUC, AP, and brier score are derived from the raw test set outputs

In contrast, the AUC, AP, and Brier score in Table 3 are fixed and robust indicators. For example, the AUC represents the area under the ROC curve, illustrating the relationship between 1-specificity and sensitivity across different thresholds. Thus, this study used AUC, AP, and Brier score as primary performance metrics, with corresponding curves displayed in Fig. 1. Specifically, the AUC values for the five models ranged from 0.793 to 0.818, with ROC curves for the test set shown in Fig. 1(A). The RF model achieved the best performance, with a Bootstrapped_ AUC of 0.917 (95% CI, 0.905–0.927) in the training set and 0.804 (95% CI, 0.787–0.818) in the test set. AP values for the five models ranged from 0.697 to 0.882, with PR curves for the test set illustrated in Fig. 1(B). The RF model also performed best in the training set, achieving an AP of 0.882 (95% CI, 0.859–0.900), while the Ridge model performed best in the test set with an AP of 0.714 (95% CI, 0.682–0.739). However, without bootstrapping in Fig. 1(B), the RF model still showed the highest AP at 0.73. Additionally, Brier scores and calibration curves in Fig. 1(C) further assess model performance, with the RF model achieving the best results, obtaining a Brier score of 0.105 in the training set and 0.170 in the test set. Overall, the RF model demonstrated the best performance among the five models.

### ML model visualization

**Fig. 2:**
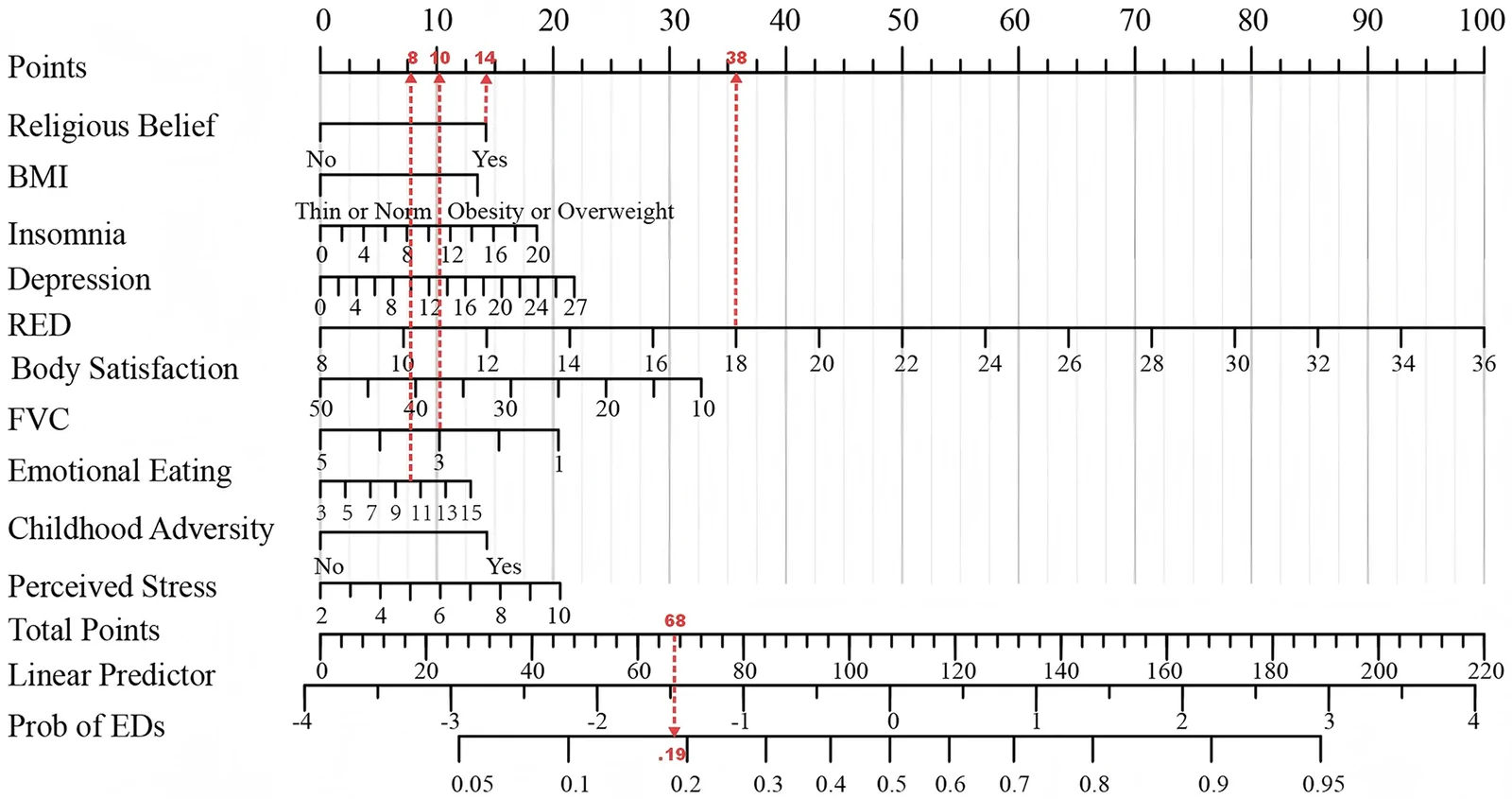
Nomogram for estimating eating disorders tendencies in Chinese nursing students (BMI: Body mass index; RED: rumination related to eating disorders; FVC: Frequency of vegetables consumption)

This study provided a nomogram in Fig. 2 for nursing educators, enabling personalized assessment of ED tendencies for each nursing student. The nomogram is a user-friendly graphical representation of a multivariate logistic regression model that visually displays the score for each risk factor. Educators can obtain a total score by summing the scores of all risk factors, which corresponds to the predicted probability of ED tendencies [41]. Notably, this tool demonstrated comparable predictive performance to the random forest model in our study, serving as a complementary tool for ML applications.

As shown in Fig. 2, a nursing student with religious beliefs (14 points), an RED score of 18 (36 points), a FVC score of 3 (10 points), and an emotional eating score of 10 (8 points), with other factors scored as 0, would have a total score of 68 points. This corresponds to a 19.11% predicted probability of ED tendencies.

To evaluate the nomogram’s calibration performance, we provided a calibration plot based on the test set (Supplementary Fig. 1). The plot showed high consistencies between the predicted and actual probability of ED tendencies in test set. Good calibration was also objectively supported by a non-significant Hosmer-Lemeshow test (*P* = 0.495 > 0.050) and a Brier score of 0.173 (acceptable threshold: <0.250) [42].

Additionally, Lasso, Ridge, and RF are non-black box algorithms that provide insights into the influence of various factors on ED tendencies, with rankings visualized in Supplementary Fig. 1. RED consistently emerged as the most significant factor, ranking first across all three models. Depression was the second most influential factor in both the Ridge and RF models. Emotional eating ranked fourth in Lasso and Ridge models and sixth in RF. Religious belief had the smallest impact on both Lasso and RF models. However, the ranking of other factors varied across the models. Childhood adversity, in particular, showed notable differences, ranking second, third, and eighth in Lasso, Ridge, and RF models, respectively.

## Discussion

In this study, the ED tendencies of 1,709 nursing students from various universities in China were assessed through an online survey. Results indicated that 39.4% of Chinese nursing students showed a high tendency toward ED, a prevalence higher than that reported among nursing students in India (28.4%; [11]), and university students in Hongkong, China (26.9%; [43]). A recent meta-analysis demonstrated that when using clinical interview-based questionnaires, such as the MINI DSM-IV and ED Diagnostic Questionnaire, as references, the SCOFF questionnaire achieved high sensitivity (0.86) and specificity (0.83) for identifying ED populations in hospital and school settings [39]. Therefore, the high ED screening rate observed in this study is likely reliable.

To our knowledge, this is the first study to identify the optimal ML algorithm for predicting ED tendencies in nursing students. Previous research has commonly relied on a single metric, the AUC, to evaluate ML model performance. In contrast, this study provides a more comprehensive assessment by including additional metrics, such as average precision (AP), Brier score, and their respective curves. RF demonstrated the highest performance among the five ML models, with 0.82 of AUC, 0.73 of AP, and 0.17 of brier score. RF, as one of the most widely used algorithms, offers several advantages, including high noise tolerance, a low risk of overfitting, and minimal requirements on dataset structure [44]. Prior studies also indicated that RF had a good performance in ED prognosis assessment. Presseller et al. [45] employed continuous glucose monitoring to predict binge eating and self-induced vomiting behaviors in ED patients, with RF achieving high accuracy rates of 0.88 and 0.79, respectively.

This study compared results from non-black box models, including Lasso, Ridge, and RF, and identified rumination related to ED as the most influential factor affecting ED tendencies across all three models, aligning with previous research findings. A recent meta-analysis indicated that rumination is moderately associated with ED in both cross-sectional and longitudinal studies [46]. Additional studies on ED among female college students have shown that heightened rumination on high-calorie foods and thin body ideals increases ED risk [47, 48]. This study also identified depression as a key factor influencing ED tendencies. A meta-analysis of longitudinal studies found that depression significantly affects the presence and severity of ED symptoms [49]. Kenny et al. [50] further suggested a bidirectional relationship between depression and ED. Clinical studies support this connection, with approximately 60% of ED patients also diagnosed with depressive disorders [51]. Moreover, this study demonstrated that body satisfaction serves as an important protective factor against ED tendencies, consistent with findings from previous research. Naeimi et al. [52] reported that medical university students with poor body image had a significantly higher risk of ED compared to those who were moderately dissatisfied or satisfied with their body image. Allen et al. [53] used a decision tree model to predict ED and found that body shape and weight concerns were the second most significant risk factor after gender.

However, our study had several findings that were inconsistent with those of previous studies. Notably, the results indicated that male sex was not a protective factor against eating disorder among Chinese nursing college students. A possible explanation is as follows: under the Chinese cultural background, the nursing profession was deemed to have low social situation and female-dominated occupation [54]; men were expected to work in male-dominated occupations with higher social status (e.g., doctor; [55]). Therefore, as Lou et al. [56] showed, male nursing students reported high life stress and moderate social support, which may have increased their risk of psychiatric disorders including eating disorder. Then, the study found religious belief was a risk factor; this contrasted with Western countries, where studies tended to consider it as a protective factor against mental diseases. Lew et al. [57] found that Christian Chinese university students had higher depression and attempted suicide risk in a large-sample investigation. The probable explanation was the “religious minority hypothesis”. In China, only a few people have religious beliefs. Due to these religious minority values, which were different compared to those main-stream groups that did not have these beliefs, it was difficult to get a sense of belonging from society. Therefore, the religious minority group suffered more psychological problems. Instead, people in Western countries with high religious affiliation constitute most of society and can more easily obtain social support from people, which protects them against mental problems. Finally, this study indicated that Chinese nursing students with a low monthly consumption expenditure had lower eating disorder risk than others with moderate or high monthly consumption level, which was inconsistent with prior study. For example, Celik et al. [58] investigated Turkish nursing college students and found that poor family economic status was associated with a higher risk of abnormal eating attitude. It was supposed that Chinese students with the lowest monthly consumption expenditure generally had poor purchase power to consume excessive food, which protected them from developing bulimic behavior [59].

The ML models can effectively and quickly screen nursing students with ED tendencies in a large sample. However, there are several limitations. Many ML algorithms are complex and not user-friendly. some only provide binary (positive or negative) outcomes, limiting the depth of information available, and they lack visual outputs that could aid nursing educators in practical application. To address these limitations, a nomogram was incorporated as a supplementary tool, offering benefits such as ease of use, visual appeal, and timeliness [60]. The nomogram in this study was based on logistic regression, demonstrating predictive performance comparable to RF.

## Limitations

Despite its strengths, this study has several limitations. First, a convenience sampling method was used to survey Chinese nursing students, primarily from Shandong Province, which may not accurately reflect the overall prevalence of eating disorders among nursing students across China. Future studies are encouraged to employ stratified sampling for broader representation. Second, although this study systematically collected many relevant influencing factors, certain biological factors (e.g., serological markers and hormone levels) could not be included due to practical constraints. This omission may have slightly reduced the ML model’s predictive performance, while it may also increase its applicability for nurse educators. Third, while the ML models were internally validated using a randomly assigned validation dataset, external validation remains the gold standard for enhancing clinical applicability [41]. Finally, regarding the application of predictive models, most ML models, including random forest (RF), are complex and not user-friendly. They typically output only binary outcomes, and lack visual outputs that could assist nursing educators in practice. Therefore, the nomogram was supplemented as a user-friendly tool, offering benefits such as ease of use, visual clarity, and timeliness. For RF, which was identified as the optimal ML model in our study, there is a learning curve to use. To address this, we have provided simplified code in Supplementary Files (ED_Prediction_Tool.ipynb, and Authors_Trained_Model.pkl) that requires only basic knowledge of file reading to operate. In the future, we may consider developing web-based interface to further improve its usability.

## Impact paragraph

In this study, multiple ML algorithms were used to construct predictive models for eating disorders (ED) tendencies among nursing students. The random forest algorithm demonstrated the highest predictive accuracy compared to the other models. Additionally, a nomogram was developed to assist educators in visually assessing individual risk factors and the likelihood of ED tendencies.

## Conclusions

This study developed ML-based predictive models to assess ED tendencies among Chinese nursing students. All non-black box models identified rumination, body satisfaction, and depression as key factors influencing ED tendencies. Among the ML algorithms tested, RF demonstrated the highest predictive performance. Additionally, a nomogram was provided as a supplementary tool, offering nursing educators a practical management aid for addressing ED tendencies.

## Relevance to educational practice

The ML predictive model enables quick and accurate screening of ED tendencies among nursing students in large samples, significantly reducing the cost for nursing educators. Additionally, this study offers evidence for ED intervention strategies, such as reducing rumination and depression and enhancing body satisfaction among nursing students. Finally, nomograms can be valuable tools for nurse educators, allowing them to provide each student with a personalized assessment of risk factors and the probability of ED tendencies, particularly in the context of psychological education for individuals showing signs of ED tendencies.

## Supplementary Information

Below is the link to the electronic supplementary material.

Supplementary Material 1

Supplementary Material 2

Supplementary Material 3

Supplementary Material 4

Supplementary Material 5

Supplementary Material 6

## Data Availability

Due to the nature of this research, participants of this study did not agree for their data to be shared publicly, so supporting data is not available.

## Abbreviations

ED: Eating Disorders
AUC: Area Under Curve
CI: Confidence Interval
AP: Average Precision
BMI: Body Mass Index
ML: Machine Learning
SVM: Support Vector Machines
RF: Random Forest
ANN: Artificial Neural Networks
SD: Standard Deviation
MD: Median
IQR: Interquartile Range
CSUL: City Size of University’s Location
HPI: History of Present Illnesses
RED: Rumination related to Eating Disorders
BCAB: Body Checking and Avoiding Behavior
TSMM: Time Spent on Meals
FHCC: Frequency of High Caloric Food Consumption
FVC: Frequency of Vegetables Consumption
FRM: Frequency of Regular Meals
CFBM: Consumption of Food Between Meals
MCE: Monthly Consumption Expenditure
CNY: Chinese Yuan
SSES: Subjective Social and Economic Status
LR: Logistic Regression
NPV: Negative Predictive Value
