# Supplementary material for: Which machine learning algorithms are best at predicting eating disorder tendencies among nursing students in China?
Source: BMC Nurs. 2026 Jun 29;25:881. doi: 10.1186/s12912-026-04951-y (PMC13602536; doi:10.1186/s12912-026-04951-y)
Supplement: Supplementary file 1 [file 12912_2026_4951_MOESM3_ESM.docx]

**Supplementary Figure 1.** Calibration plot in test set (N = 513) for eating disorders tendencies among Chinese nursing students.

**Supplementary Figure 2.** The features rank of non-black box machine learning models (BMI: Body mass index, RED: Rumination related to eating disorders, FVC: Frequency of vegetables consumption)
