# Supplementary material for: Which machine learning algorithms are best at predicting eating disorder tendencies among nursing students in China?
Source: BMC Nurs. 2026 Jun 29;25:881. doi: 10.1186/s12912-026-04951-y (PMC13602536; doi:10.1186/s12912-026-04951-y)
Supplement: Supplementary file 2 [file 12912_2026_4951_MOESM4_ESM.docx]

**Supplementary Table 1.1** Questionnaires’ name, author, years, item number, score range, internal reliability, and model fit.

| Variables | Questionnaire (Authors, Years) | Items | Score Range | Cronbach's α | χ^2^/df | *P* | CFI | TLI | RMSEA |
| --- | --- | --- | --- | --- | --- | --- | --- | --- | --- |
| Eating Disorder | Sick Control Stone Fat Food Questionnaire (Morgan et al., 1999) | 5 | 0-5 | 0.536 | 2.8635 | <0.05 | 0.983 | 0.957 | 0.033 |
| Insomnia | Athens Insomnia Scale (Soldatos et al., 2000) | 8 | 0-24 | 0.842 | 7.0045 | <0.05 | 0.953 | 0.934 | 0.066 |
| Impulsivity | Brief Barratt Impulsiveness Scale (Steinberg et al., 2013) | 8 | 4-32 | 0.838 | 6.8112 | <0.05 | 0.981 | 0.971 | 0.059 |
| Perfectionism | Adapted Frost Multidimensional Perfectionism Scale (Burgess et al., 2016) | 6 | 5-30 | 0.795 | 10.2294 | <0.05 | 0.987 | 0.971 | 0.074 |
| Anxiety | Generalized Anxiety Disorder Scale (Spitzer et al., 2006) | 7 | 0-21 | 0.936 | 5.8526 | <0.05 | 0.988 | 0.982 | 0.054 |
| Depression | Patient Health Questionnaire (Kroenke et al., 2001) | 9 | 0-27 | 0.940 | 4.4741 | <0.05 | 0.987 | 0.983 | 0.045 |
| Emotional Eating | Intuitive Eating Subscale (Tylka & Kroon Van Diest, 2013) | 3 | 3-15 | 0.837 | 0.0000 | <0.05 | 1.000 | 1.000 | 0.000 |
| Rumination | Ruminative Response Scale for Eating Disorder (Cowdrey & Park, 2011) | 9 | 9-36 | 0.926 | 10.6127 | <0.05 | 0.969 | 0.953 | 0.075 |
| Body Satisfaction | Multidimensional Body Self-Relations Subscale (Brown et al., 1990) | 9 | 9-45 | 0.908 | 5.1479 | <0.05 | 0.981 | 0.974 | 0.050 |
| Body Checking Behavior | Body Checking and Avoidance Questionnaire (Shafran et al., 2004; Adapted Version) | 5 | 5-25 | 0.880 | 6.7065 | <0.05 | 0.993 | 0.983 | 0.058 |
| Exercise | Physical Activity Rating Scale (Li & Xia, 2021) | 3 | 0-100 | 0.626 | 0.0000 | <0.05 | 1.000 | 1.000 | 0.000 |
| Social Support | Oslo Social Support Scale (Kocalevent et al., 2018) | 3 | 3-14 | 0.717 | 0.0000 | <0.05 | 1.000 | 1.000 | 0.000 |
| Loneliness | Three-Item Loneliness Scale (Hughes et al., 2004) | 3 | 3-9 | 0.804 | 0.0000 | <0.05 | 1.000 | 1.000 | 0.000 |
| Childhood Adversity | Adverse Childhood Experience Questionnaire (Finkelhor et al., 2015) | 9 | 0-9 | 0.874 | 4.095 | <0.05 | 0.946 | 0.928 | 0.043 |
| Perceived Stress | Perceived Stress Scale (Herrero & Meneses, 2006) | 2 | 2-10 | 0.700 | - | - | - | - | - |
| Introversion | Big-Five Inventory (BFI-10) (Carciofo et al., 2016) | 2 | 2-10 | 0.281 | - | - | - | - | - |
| Neuroticism | Big-Five Inventory (BFI-10) (Carciofo et al., 2016) | 2 | 2-10 | 0.263 | - | - | - | - | - |
| Conscientiousness | Big-Five Inventory (BFI-10) (Carciofo et al., 2016) | 2 | 2-10 | 0.344 | - |  |  | - | - |
| Socioeconomic Status | Subjective Socioeconomic Status Scale (Adler et al., 2000) | 1 | 1-10 | - | - | - | - | - | - |

**Notes:** Cronbach’s α ≥0.70 is adequately acceptable, 0.60-0.70 marginally acceptable but requires cautious interpretation, <0.60 unacceptable ; χ^2^/df <12.000, CFI>0.900, TLI>0.900, RMSEA<0.080 indicates good construct validity of the questionnaires; only the item number ≥ 3 could be conducted confirmatory factor analysis for obtaining questionnaires’ construct validity. The SCOFF, a widely used EDs screening tool, typically showed lower Cronbach’s α due to low inter-item correlations among its five core items, and was generally reported between 0.40 and 0.60 in previous studies (Bazzazian et al., 2022, Beer et al., 2025). The two-item personality questionnaire’s low Cronbach’s α was due to its limited items and reverse scoring (Ellen et al., 2022, Suárez-Álvarez et al., 2019).

**Supplementary Table 1.2** The details of questionnaire’s item content, scoring method.

[1]. Eating Habits Questionnaire (EHQ-5)

| 1. Time spent on main meals | □ <10min; □ 10-15min; □15-30min; □30-60min; □ >1h |
| --- | --- |

| Item | Never | Occasionally | Sometimes | Often | Always |
| --- | --- | --- | --- | --- | --- |
| 2. Frequency of consumption of high calorie foods (e.g., fried foods, sugary foods, etc.) | |  |  |  |  |
| 3. Frequency of vegetable consumption |  |  |  |  |  |
| 4. Frequency of regular meals (that is, eating at a fixed time) |  |  |  |  |  |
| 5. Frequency of foods between meals |  |  |  |  |  |

[2]. Body Checking and Avoidance Behavior Questionnaire (BCAB)

| Item | Never (Uninterested) | | | 1-3times/week | 1-3times/day | | | ≥ 4 times/day | Never (Overconcerned) |
| --- | --- | --- | --- | --- | --- | --- | --- | --- | --- |
| 1. Look or touch your body areas, such as face, belly, bottom, and thighs |  | | |  |  | | |  |  |
| 2. Look in mirror at your body areas, such as  face, belly, bottom, and thighs |  | | |  |  | | |  |  |
| 3. Compare your body areas with others, such as face, belly, bottom, and thighs |  | | |  |  | | |  |  |
| 4. Use a tape measure around your body areas, such as face, belly, bottom, and thighs |  | | |  |  | | |  |  |
| 5. Use a scale weighing yourself | |  |  | | |  |  | |  |

**Supplementary Table 2**. The machine learning models’ training results and predictive performance in training and test set.

| Model | Training Results | AUC | |
| --- | --- | --- | --- |
|  |  | Training Set | Test set |
| Lasso | LogisticRegression(C =1000.0, solver=‘liblinear’, penalty = ‘l1’, max_iter = 5000, random_state=42) | 0.7982949 | 0.792652 |
| Ridge | LogisticRegression(C = 0.0227697025538168, solver=‘liblinear’, penalty = ‘l2’, max_iter = 5000, random_state=42) | 0.7991697 | 0.797971 |
| RF | RandomForestClassifier(criterion=‘entropy’, n_estimators=377,min_samples_split=17, min_samples_leaf=1, max_depth=14, random_state=42) | 0.8080469 | 0.817700 |
| SVM | SVC(C=0.014174741629268055, kernel=‘linear’, max_iter = 5000, random_state=42, probability=True) | 0.7979199 | 0.794561 |
| ANN | MLPClassifier(alpha=1.629750834620645, learning_rate=‘constant’, hidden_layer_sizes=13, activation=‘identity’, solver=‘sgd’, max_iter=5000, random_state=42) | 0.7994077 | 0.794776 |

**Notes:** RF: Random Forest; SVM: Support Vector Machine; ANN: Artificial Neural Network; AUC: Area Under Curves of ROC; The AUC of the training set is the average after 10 fold cross-validation.

REFERENCE

Bazzazian, S., Ozgoli, G., Kariman, N., Nasiri, M., Mokhtaryan-Gilani, T., & Hajiesmaello, M. (2022). The translation and psychometric	assessment of the SCOFF eating disorder screening questionnaire: the Persian version. *Journal of Eating Disorders*, *10(1), 38.	<https://doi.org/10.1186/s40337-022-00564-3>*

Beer, C. S., Lüken, L. M., Eggendorf, J., Holtmann, M., & Legenbauer, T. (2025). Validation of the SCOFF as a simple screening tool for eating disorders in an inpatient sample before and during COVID-19. International Journal of Eating Disorders, 58(1), 243-247. https://doi.org/10.1002/eat.24318

Brown, T. A., Cash, T. F. in Mikulka, P. J. (1990). Attitudinal Body-Image Assessment: Factor Analysis of the Body-Self Relations Questionnaire. *Journal of Personality Assessment*, *55*(1-2), 135-144. https://doi.org/10.1080/00223891.1990.9674053

Burgess, A. M., Frost, R. O. in DiBartolo, P. M. (2016). Development and Validation of the Frost Multidimensional Perfectionism Scale–Brief. *Journal of Psychoeducational Assessment*, *34*(7), 620-633. https://doi.org/10.1177/0734282916651359

Cowdrey, F. A. in Park, R. J. (2011). Assessing rumination in eating disorders: Principal component analysis of a minimally modified ruminative response scale. *Eating Behaviors*, *12*(4), 321-324. https://doi.org/10.1016/j.eatbeh.2011.08.001

Ellen, B. P., Mackey, J. D., McAllister, C. P., & Mercer, I. S. (2022). Are small measures big problems? A meta-analytic investigation of brief measures of the Big Five. Journal of Business Research, 151, 579-592. https://doi.org/10.1016/j.jbusres.2022.07.027

Finkelhor, D., Shattuck, A., Turner, H. in Hamby, S. (2015). A revised inventory of Adverse Childhood Experiences. *Child Abuse & Neglect*, *48*, 13-21. https://doi.org/10.1016/j.chiabu.2015.07.011

Herrero, J. in Meneses, J. (2006). Short Web-based versions of the perceived stress (PSS) and Center for Epidemiological Studies-Depression (CESD) Scales: a comparison to pencil and paper responses among Internet users. *Computers in Human Behavior*, *22*(5), 830-846. https://doi.org/10.1016/j.chb.2004.03.007

Hughes, M. E., Waite, L. J., Hawkley, L. C. in Cacioppo, J. T. (2004). A Short Scale for Measuring Loneliness in Large Surveys. *Research on Aging*, *26*(6), 655-672. https://doi.org/10.1177/0164027504268574

Kocalevent, R.-D., Berg, L., Beutel, M. E., Hinz, A., Zenger, M., Härter, M.et al. (2018). Social support in the general population: standardization of the Oslo social support scale (OSSS-3). *BMC Psychology*, *6*(1). https://doi.org/10.1186/s40359-018-0249-9

Kroenke, K., Spitzer, R. L. in Williams, J. B. W. (2001). The PHQ-9: validity of a brief depression severity measure. *Journal of General Internal Medicine*, *16*(9), 606-613. https://doi.org/10.1046/j.1525-1497.2001.016009606.x

Li, X.-l. in Xia, M. (2021). The Positive Effect of Perceived Exercise Benefit and the Negative Effect of Perceived Severity of Disease and Weakness on College Students' Amount of Exercise: The Mediate and Suppressor Role of Physical Fitness Evaluation Self-Efficacy. *Frontiers in Psychology*, *12*. https://doi.org/10.3389/fpsyg.2021.762865

Morgan, J. F., Reid, F. in Lacey, J. H. (1999). The SCOFF questionnaire: assessment of a new screening tool for eating disorders. *Bmj*, *319*(7223), 1467-1468. https://doi.org/10.1136/bmj.319.7223.1467

Qiu, J., Carciofo, R., Yang, J., Song, N., Du, F. in Zhang, K. (2016). Psychometric Evaluation of Chinese-Language 44-Item and 10-Item Big Five Personality Inventories, Including Correlations with Chronotype, Mindfulness and Mind Wandering. *Plos One*, *11*(2). https://doi.org/10.1371/journal.pone.0149963

Shafran, R., Fairburn, C. G., Robinson, P. in Lask, B. (2003). Body checking and its avoidance in eating disorders. *International Journal of Eating Disorders*, *35*(1), 93-101. https://doi.org/10.1002/eat.10228

Soldatos, C. R., Dikeos, D. G. in Paparrigopoulos, T. J. (2000). Athens Insomnia Scale: validation of an instrument based on ICD-10 criteria. *Journal of Psychosomatic Research*, *48*(6), 555-560. https://doi.org/10.1016/s0022-3999(00)00095-7

Spitzer, R. L., Kroenke, K., Williams, J. B. W. in Löwe, B. (2006). A Brief Measure for Assessing Generalized Anxiety Disorder. *Archives of Internal Medicine*, *166*(10). https://doi.org/10.1001/archinte.166.10.1092

Steinberg, L., Sharp, C., Stanford, M. S. in Tharp, A. T. (2013). New tricks for an old measure: The development of the Barratt Impulsiveness Scale–Brief (BIS-Brief). *Psychological Assessment*, *25*(1), 216-226. <https://doi.org/10.1037/a0030550>

Suárez-Álvarez, J., Pedrosa, I., Lozano, L. M., García-Cueto, E., Cuesta, M., & Muñiz, J. (2019). Using reversed items in Likert scales: A questionable practice. Psicothema, 30(2), 149–158. https://doi.org/10.7334/psicothema2018.165

Tylka, T. L. in Kroon Van Diest, A. M. (2013). The Intuitive Eating Scale–2: Item refinement and psychometric evaluation with college women and men. *Journal of Counseling Psychology*, *60*(1), 137-153. https://doi.org/10.1037/a0030893
